# Safe and Efficacious Use of Low‐Dose Rituximab in Postpubertal Paediatric Patients With Immune Thrombocytopenia

**DOI:** 10.1002/jha2.70010

**Published:** 2025-05-01

**Authors:** Jennifer Darlow, Gerard Gurumurthy, Nathan Jeffreys, Lianna Reynolds, Vismay Deshani, John Grainger

**Affiliations:** ^1^ Royal Manchester Children's Hospital Manchester UK; ^2^ University of Manchester Manchester UK

## Abstract

**Background:**

Rituximab, a CD20 monoclonal antibody, is used in chronic/refractory immune thrombocytopenia (ITP). Standard dosing is 375 mg/m^2^ weekly for 4 weeks alongside dexamethasone. A lower dose of Rituximab at 100 mg weekly demonstrates comparable efficacy that is well tolerated. This study evaluates lower‐dose Rituximab with dexamethasone in postpubertal pediatric ITP patients.

**Methods:**

Patients treated with 100 mg weekly Rituximab for 4 weeks alongside dexamethasone at 10 mg/m^2^ on days 1–5 and 21–25 were assessed for response and safety.

**Results:**

Of the 10 patients treated, six responded completely, one partially and three showed no response. Four responders maintained their response over 2 years. One Rituximab‐related infusion reaction and one Dexamethasone‐related adverse event were reported.

**Conclusion:**

Rituximab 100 mg weekly may be non‐inferior to 375 mg/m^2^ weekly for paediatric patients.

## 1 Introduction

Immune thrombocytopenia (ITP) is an autoimmune condition characterised by isolated thrombocytopenia in the absence of other causes [1]. The exact pathogenesis of ITP is unclear. However, it involves pathogenic anti‐platelet antibodies, T cell‐mediated destruction and impaired megakaryocyte function [2]. The clinical manifestation of ITP is variable, from asymptomatic to life‐threatening bleeding. Haematologists balance the risk of significant bleeding complications and direct treatment accordingly [3].

Management strategies for ITP in paediatrics aim to reduce bleeding risk. First‐line treatment is with immunomodulation with corticosteroids and intravenous immunoglobulins (IVIg). Corticosteroids, such as prednisone, achieve an initial response in up to 80% of cases [4]. Despite this, the durability of response following tapering of corticosteroids is relatively low, with sustained response observed in only up to 50% of patients [4].

Alternative therapies for individuals who are unresponsive to or dependent on corticosteroid treatment are diverse and are selected based on patient‐specific factors including age, disease severity and response to previous treatments [5]. Historical options like splenectomy, while associated with long‐term response rates of up to 70%, have seen a decline in utilisation in favour of novel pharmacological treatments [6]. Newer agents, like thrombopoietin receptor agonists, offer similarly high response rates at around 75% [7].

The role of B cells in the pathogenesis of ITP has been well established [8]. Rituximab, an anti‐CD20 chimeric monoclonal antibody, mediates B cell depletion thereby reducing the production of autoantibodies against platelets [8]. The International Working Group on ITP recommends Rituximab as a second‐line therapy, with a regimen of 375 mg/m^2^ weekly for 4 weeks [1]. This regimen has demonstrated an overall response rate (ORR) of 69% with a complete response (CR) in 54% of adults [9]. Bussel et al. demonstrated a sustained response in 41% of children (median age: 12, range: 1–17), limited to the adolescent female population [10]. The addition of dexamethasone to Rituximab therapy aims to synergise with this treatment by leveraging the anti‐plasma effects of dexamethasone [11].

Despite the established benefits of the standard 375 mg/m^2^ regimen of rituximab, emerging evidence suggests that a lower dose regimen (100 mg weekly for 4 weeks) could offer comparable efficacy [12, 13]. This low‐dose approach has shown a similar overall initial response rate to the standard dose but with potentially fewer adverse events and lower costs.

Studies exploring the low‐dose Rituximab regimens in the paediatric population are scarce and there remains a substantial gap in understanding the efficacy and safety of the 100 mg dosage in younger patients with ITP. This short report examines the use of 100 mg Rituximab in conjunction with low‐dose dexamethasone in post‐pubertal paediatric patients, exploring both clinical outcomes and safety profiles in this specific group.

## 2 Methods

### 2.1 Study Design and Patient Selection

This service evaluation was conducted at the Royal Manchester Children's Hospital, Manchester, UK, where data from post‐pubertal paediatric patients diagnosed with ITP and treated with Rituximab were analysed. Patients included in the study were those treated between January 2010 and May 2024 who received a regimen of low‐dose Rituximab (100 mg weekly for 4 weeks). Consent for the use of clinical data was obtained from all participants or their legal guardians as part of their enrolment in the national ITP registry and for service evaluation.

### 2.2 Treatment Protocol

Patients received Rituximab at a dose of 100 mg administered intravenously once weekly for four consecutive weeks. Adjunctive treatment with dexamethasone was provided in line with local protocol. The dosing regimen of dexamethasone was adopted from the UKALL 2019 guidelines at 10 mg/m^2^ on days 1–5 and 21–25 of the treatment cycle.

### 2.3 Data Collection

Data was collected from patient's electronic and paper health records. Key variables included demographic information (age, sex), duration of ITP prior to Rituximab treatment and response to prior therapies. Clinical outcomes measured were platelet counts at baseline and 4, 8 and 12 weeks post‐initiation of Rituximab therapy. Response to treatment was categorised as a complete response (platelet count >100 × 10^9^/L) or partial response (platelet count >30 × 10^9^/L but <100 × 10^9^/L) at 3 months following treatment initiation.

### 2.4 Outcome Measures

The primary outcome measure was the response rate to Rituximab, defined by the achievement of either a complete or partial response at 12 weeks. Secondary outcomes included the duration of response, measured as the time from treatment initiation to the first recorded platelet count below the response threshold or the need for additional ITP‐specific therapy. Incidences of any adverse events or side effects attributed to Rituximab and Dexamethasone were also noted.

## 3 Results

### 3.1 Patient Demographics and Baseline Characteristics

A total of 10 patients aged between 10 and 17 years (median age: 13.8 years) met the inclusion criteria for this study (Data S1). The cohort comprised six females and four males. The median time from ITP diagnosis to the start of Rituximab treatment was 19.0 months (interquartile range [IQR]: 8.3–23.0). The median follow‐up for the overall series was 116.6 months (IQR: 45.7–300.4)

### 3.2 Treatment Response

**FIGURE 1:**
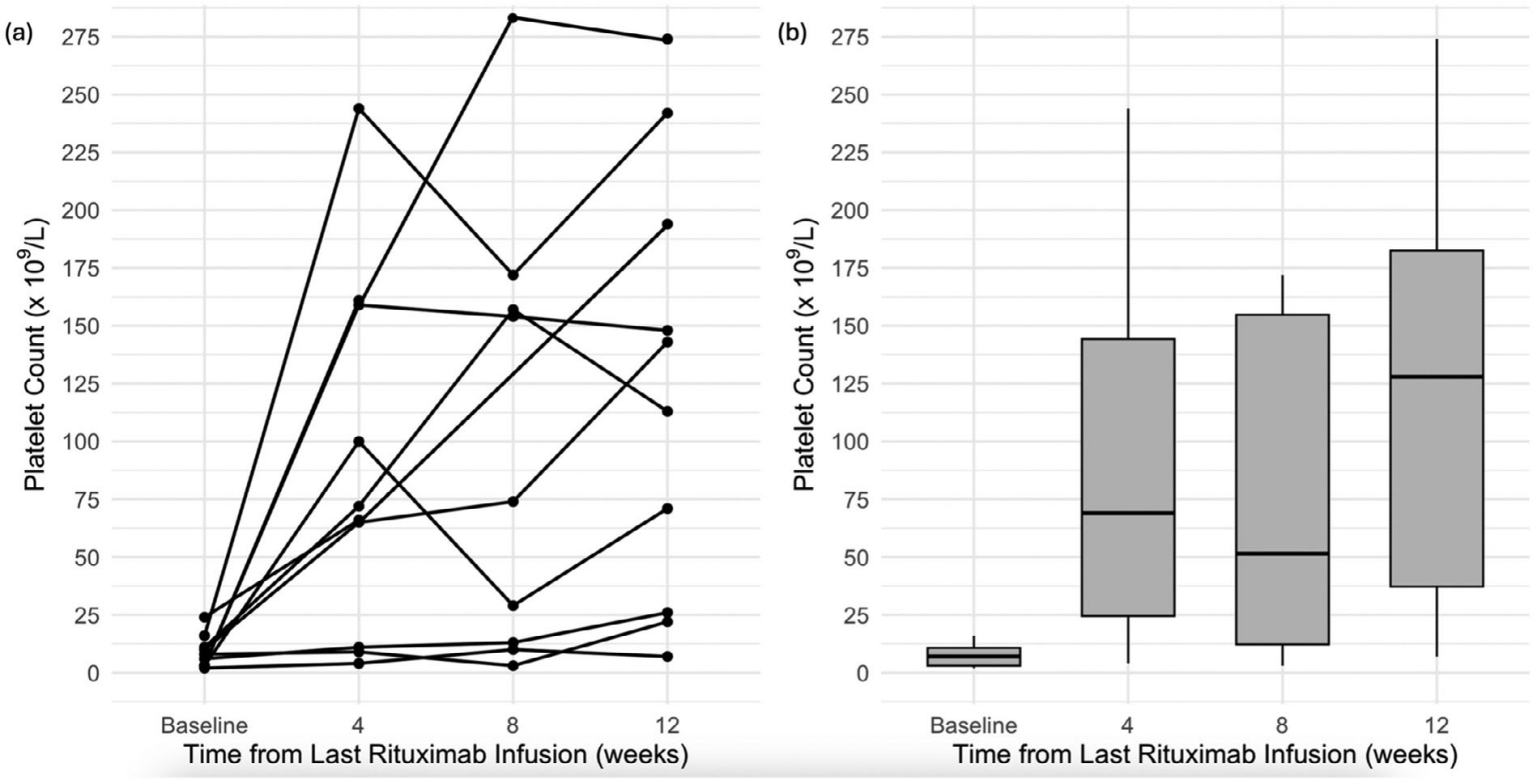
(a) Individual patient platelet counts over time from the last Rituximab infusion. (b) Median platelet count over time from last Rituximab infusion.

**FIGURE 2:**
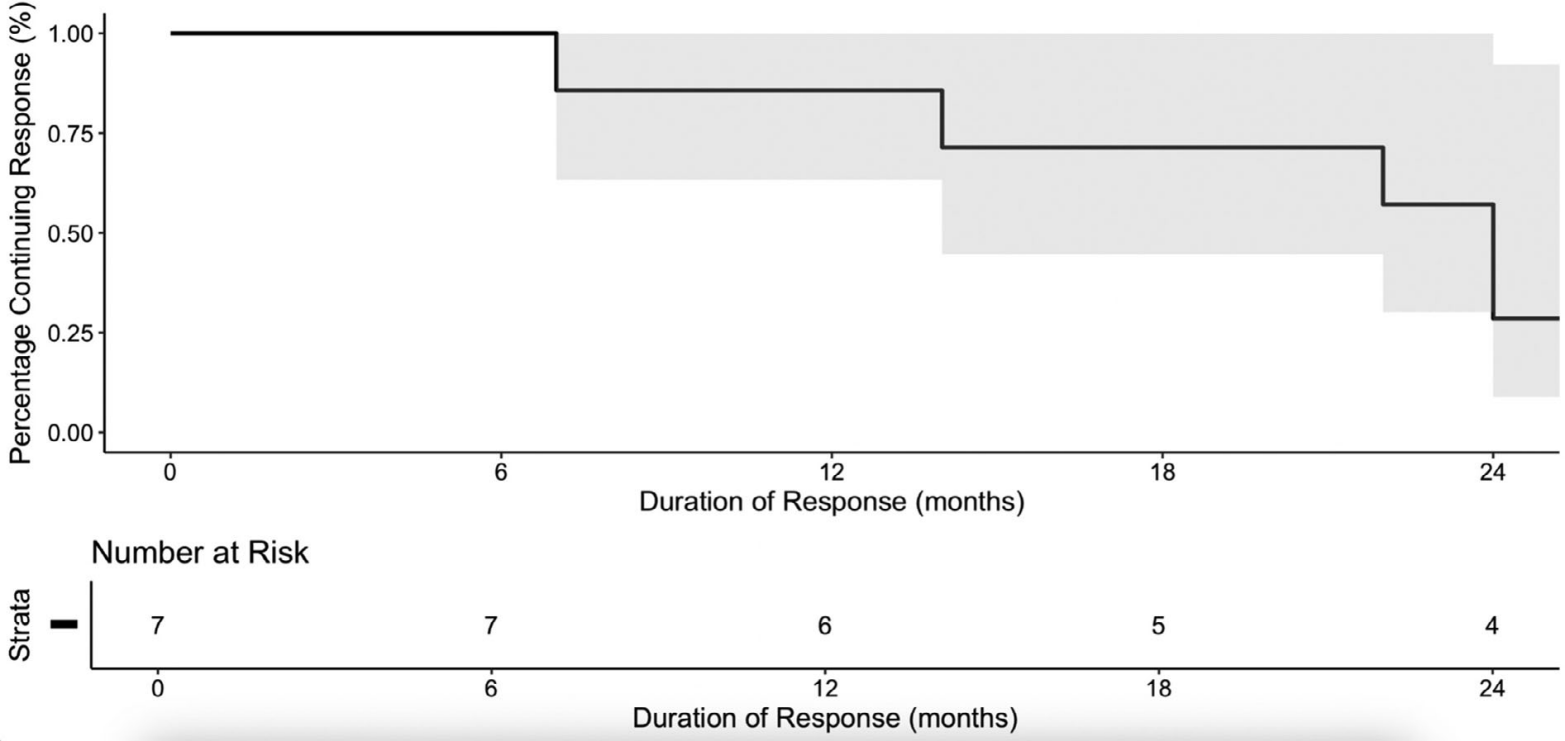
Kaplan‐Meier analysis of duration of response to lower dose Rituximab (100 mg). Four out of 10 patients maintained their response over 2 years.

Six patients (60%) achieved a complete response and one patient (10%) exhibited a partial response at 12 weeks (Figure 1). Three patients (30%) showed no significant increase in platelet counts and were classified as non‐responders. The ORR for the cohort was 70%, with a CR of 60%. Four out of six females had responded at 3 months follow‐up compared to three out of four males. The ages of the seven responders varied from 11–16 years, with a median age of 14.9 years (IQR: 13.7–15.1). The duration of sustained response ranged from 6 months to 14 years, with a median duration of 22 months (IQR: 10.5–90 months). Four out of seven responders (three female and one male) maintained their response for more than 2 years post‐treatment (Figure 2). The remaining three responders had varying response durations of 7–22 months, with a median duration of 14.0 months (IQR: 10.5–18.0). These individuals went on to receive further treatments outlined in Data S1. One of these individuals received a second course of Rituximab but at the standard dose of 375 mg/m^2^, 20.5 years after the initial treatment, at the age of 29. This led to a complete response. At the most recent follow‐up 3 years later, the patient's platelet count remained above 100 × 10⁹/L.

### 3.3 Adverse Events and Safety

One patient had an infusion reaction to Rituximab during the first cycle. This was managed by pausing the infusion and administering intravenous hydrocortisone. The patient subsequently completed a further three cycles with no further events. No other side effects attributable to Rituximab were reported in the cohort. One patient reported dexamethasone‐related side effects, including acne, mood swings and changes in appetite.

## 4 Discussion

The findings indicate a 70% ORR with 60% of patients achieving a CR which is comparable to the efficacy reported for initial response in the standard‐dose regimen of rituximab (375 mg/m^2^ weekly for 4 weeks) in adult ITP populations. This correlates with previous studies, which have demonstrated that the initial response in the standard dose regimen yields an ORR of approximately 69%, with a CR of 54%​​ [9]. Bussel et al. reported a higher likelihood of sustained responses in female children in their study at standard doses [10]. We found no significant difference in sustained response rates between genders, although this could be due to the small sample size of this study. In the paediatric population, a systematic review of standard‐dose Rituximab determined the ORR and CR rates were approximately 68% and 39% respectively, suggesting similar efficacy to the low‐dose Rituximab achieved in this study [14].

A meta‐analysis in the adult population reported comparable CR, ORR and sustained response rates between low‐dose and standard‐dose regimens, suggesting that lower doses might be an effective alternative [12]. In our cohort, four patients maintained their response for over 2 years, highlighting the potential for durable responses with the lower dose regimen. Notably, one patient received a second course of Rituximab at a standard dose. His initial response lasted under 2 years, which was not classified as a durable response to the initial therapy. The subsequent re‐administration of Rituximab at standard dose, however, achieved a complete response that has been sustained for 3 years and is ongoing.

An advantage of low‐dose rituximab is that it confers a lower risk of iatrogenic adverse effects as both infection and acute infusion reactions are dose‐dependent [15]. Rituximab is associated with hypogammaglobulinaemia and increased infections. One meta‐analysis comparing low‐dose and standard‐dose Rituximab in the adult population identified a trend toward lower infection rates in the low‐dose Rituximab group [12]. In our small, non‐comparative paediatric cohort, no infections were observed following low‐dose rituximab. However, given the limited sample size and the fact that these data are derived primarily from adult studies, it is not possible to draw definitive conclusions regarding infection risk reduction in children.

Rituximab is also associated with infusion reactions. These are more likely on the first infusion and are considered dose and rate‐dependent [15]. One infusion reaction was reported in the 10 patients in this case series. This data supports the theory that a low‐dose Rituximab regimen enhances patient safety.

Data in a paediatric population is limited by low numbers treated with Rituximab. Although the small sample size limits the statistical power and generalisability of the findings in this case series, it correlates with the results of larger adult data sets.

## 5 Conclusion

The study highlights the potential of the low‐dose regimen of rituximab (100 mg weekly combined with dexamethasone) as a safe and effective second‐line treatment for paediatric patients with ITP. Given the comparable efficacy to standard‐dose regimens and the improved safety profile, this treatment approach warrants further clinical evaluation.

## Author Contributions

Jennifer Darlow, Nathan Jeffreys, Vismay Deshani and Gerard Gurumurthy collected and analysed the data. Gerard Gurumurthy, Lianna Reynolds and John Grainger wrote the manuscript. All authors reviewed the manuscript.

## Conflicts of Interest

The authors declare no conflicts of interest.

## Ethics Approval

Ethics approval was not required for this study as patient consent was obtained for the use of their data for service evaluations.

## Consent

The authors have nothing to report.

## Clinical Trial Registration

Not applicable.

## Supporting information




Supporting Information

## Data Availability

All data supporting the findings of this study are available within the paper.
