# Supplementary material for: Safe and Efficacious Use of Low‐Dose Rituximab in Postpubertal Paediatric Patients With Immune Thrombocytopenia
Source: EJHaem. 2025 May 1;6(3):e70010. doi: 10.1002/jha2.70010 (PMC12044411; doi:10.1002/jha2.70010)
Supplement: Supplementary file 1 — Supporting Information [file JHA2-6-e70010-s001.docx]

**Supplementary Data S1. Patient characteristics, response to Rituximab and treatment-related side effects.**

| **Patient No.** | **Disease Status** | **Gender** | **Previous Treatment** | **Age on First Infusion (years)** | **Platelet Count at Baseline (10^9^/L)** | **Platelet Count at 4 weeks (10^9^/L)** | **Platelet Count at 8 weeks (10^9^/L)** | **Platelet Count at 12 weeks (10^9^/L)** | **Duration of Response (months)** | **Need for Rescue Treatment after Rituximab** | **Further Treatment after Rituximab** | **Treatment-related Side Effects** |
| --- | --- | --- | --- | --- | --- | --- | --- | --- | --- | --- | --- | --- |
| 1 | Primary ITP | Female | Romiplostim, MMF, IVIg & Steroids | 16 | 3 | 161 | 282 | 274 | > 24 | No | None | None reported |
| 2 | Primary ITP | Female | Romiplostim, IVIg & Steroids | 14 | 11 | 72 | 157 | 113 | 14 | No | None | None reported |
| 3 | Secondary ITP  (Evans Syndrome) | Female | Sirolimus, Prednisolone, Romiplostim | 10 | 8 | 9 | 3 | 22 | NA | No | None | None reported |
| 4 | Primary ITP | Female | IVIg, Steroids | 15 | 16 | 244 | 172 | 242 | > 24 | No | None | None reported |
| 5 | Primary ITP | Male | Steroids | 7 | 2 | 4 | 10 | 7 | NA | No | Romiplostim (CR) | None reported |
| 6 | Primary ITP | Female | Steroids, IVIg, Romiplostim | 11 | 10 | 65 | 74 | 143 | > 24 | No | None | None reported |
| 7 | Primary ITP | Male | Steroids, IVIg | 13 | 3 | 159 | 154 | 148 | 22 | Yes - steroids | MMF (PR)  2^nd^ Rituximab (CR) | None reported |
| 8 | Primary ITP | Male | Steroids | 15 | 24 | 66 | - | 194 | > 24 | No | None | None reported |
| 9 | Primary ITP | Female | None* | 14 | 6 | 11 | 13 | 26 | NA | No | Avatrombopag (CR) | Dexamethasone-related: mood, acne and appetite |
| 10 | Primary ITP | Male | Steroids | 12 | 3 | 100 | 29 | 71 | 7 | Yes - steroids | Steroids (PR) | Rituximab-related: infusion reaction |

* This patient received Rituximab as a first-line treatment due to familial and social circumstances/ preference for less follow-ups.

Abbreviations: CR = complete response, defined as platelet count > 100 × 10^9^/L; IVIg = immunoglobulins; MMF = Mycophenolate mofetil; PR = partial response, defined as platelet count > 30 × 10^9^/l but <100 × 10^9^/L
